# Yes‐associated protein promotes tumour necrosis factor α–treated cementoblast mineralization partly by inactivating NF‐κB pathway

**DOI:** 10.1111/jcmm.15426

**Published:** 2020-06-08

**Authors:** Lu Zhang, Hualing Sun, Jing Zhang, Fangfang Song, Liyuan Huang, Zhengguo Cao, Cui Huang

**Affiliations:** ^1^ The State Key Laboratory Breeding Base of Basic Science of Stomatology (Hubei‐MOST) & Key Laboratory of Oral Biomedicine Ministry of Education, School & Hospital of Stomatology Wuhan University Wuhan China; ^2^ Department of Periodontics Yantai Stomatological Hospital Yantai China

**Keywords:** cementoblast, cementogenesis, inflammation, NF‐κB pathway, TNF‐α, YAP

## Abstract

Cementum regeneration, as one of the most difficult challenges of periodontal regeneration, is influenced by inflammatory factors. Inflammation may hamper or promote periodontal tissue repair under different circumstances, as it is found to do in dentin‐pulp complex and bone tissue. Our team demonstrated that YAP promotes mineralization of OCCM, a cementoblast cell line. However, the effect of YAP on its mineralization under inflammatory microenvironment is unclear. In this study, cementogenesis in vitro was up‐regulated after transient TNF‐α treatment for 30 minutes. YAP expression also was increased by TNF‐α treatment. YAP overexpression promoted OCCM mineralization after the cells were transiently treated with TNF‐α because YAP overexpression inhibited NF‐κB pathway activity, while YAP knockdown elevated it. The inhibited mineralization potential and activated NF‐κB pathway activity by YAP knockdown also were partly rescued by the application of the NF‐κB inhibitor Bay 11‐7082. These results demonstrated that YAP plays a positive role in the mineralization of TNF‐α transiently treated cementoblast, partly by inhibiting the NF‐κB pathway activity.

## 1 INTRODUCTION

Cementoblast serves as a crucial cell to maintain cementum homeostasis, which is an essential part of periodontal homeostasis. Inflammatory factors, which are highly concentrated in tissues affected by periodontitis, are often found to undermine cementum and periodontal homeostasis by suppressing the function of cementoblast in addition to those of osteoblast and osteoprogenitors.^1^, ^2^, ^3^ Recently, a review suggests that inflammation is a double‐edged sword in the regeneration of the dentin‐pulp complex. Low inflammation levels may promote mineralized tissue repair, whereas persistent chronic inflammation inhibits it.^4^ We wondered how cementoblast would work under inflammatory factor treatment with limited duration. This knowledge would provide insight into the mechanism by which the mineralization of cementoblast is regulated in inflammatory microenvironment and is significant for cementum homeostasis maintenance.

YAP is a transcription coactivator that plays a role in cell proliferation and differentiation.^5^ With regard to mineralization, YAP has varied effects in different cell types. YAP transcriptional activity in the endothelial cells of zebrafish promotes intramembranous ossification.^6^ In cementoblast cell line, YAP promotes mineralization by regulating BMP/Smad and Erk1/2.^7^

However, the effect of YAP on cementoblast mineralization in inflammatory microenvironment is rarely documented. In this study, we aimed to determine the role of YAP in inflammation‐influenced cementoblasts.

NF‐κB is a central mediator of signal transduction in inflammatory response.^8^ From most published works, NF‐κB was shown to negatively regulate mineralization of osteoblast and other cells.^9^, ^10^, ^11^ To our knowledge, few works have studied the effect of YAP on the NF‐κB pathway, and this effect is reasonably related to how YAP regulates cementoblast mineralization.

In the present study, the mineralizing potential and YAP expression of an immortalized cementoblast OCCM‐30 treated by TNF‐α were investigated. Then, after transiently treated with TNF‐α, the NF‐κB pathway activity and the mineralization of OCCM‐30 with overexpressed and knockdown YAP was evaluated. Finally, rescuing experiments were performed to reveal the role of the NF‐κB pathway in the YAP‐regulated mineralization of OCCM‐30 that was transiently treated with TNF‐α.

## 2 MATERIALS AND METHODS

### 2.1 Ethical statement

All experiments were approved by the Institutional Ethical Board of Wuhan University and were done followed by the guidelines of the National Institute of Health (NIH).

### 2.2 Periodontal tissue inflammation induction and tissue section preparation

Apical periodontal lesion of mice was induced as reported previously.^12^ After cervical dislocation, the animals were ready for mandible harvest. The mandibles were fixed in 4% paraformaldehyde overnight and then immersed in 10% ethylenediaminetetraacetic acid (EDTA; pH of 7.2) at room temperature for 3 weeks. The mandibles were embedded in paraffin and sectioned longitudinally in the mesiodistal direction at the apex level of the treated tooth.

### 2.3 Cell culture and mineralization induction

An immortalized mouse cementoblast OCCM‐30 generously provided by Dr Martha J. Somerman was used in this study. The cells were cultured in Dulbecco's modified Eagle's medium (Hyclone) supplemented with 10% (*v/v*) foetal bovine serum (Biological Industries, Israel) and 1% (*v/v*) penicillin‐streptomycin in 5% CO_2_ and 37°C. When the seeded cells reached 80% confluence, TNF‐α was added into the wells to different concentrations. Then, the effect of TNF‐α on the YAP protein expression was evaluated. To induce mineralization, we pre‐treated the OCCM of 80% confluence with 10 ng/mL TNF‐α for 30 minutes. Then, the medium was changed into a mineralization medium containing 50 µg/mL L‐ascorbic acid (Sigma‐Aldrich) and 10 mmol/L β‐glycerophosphate disodium salt hydrate (Sigma‐Aldrich).

### 2.4 Immunofluorescence staining

YAP protein localization in OCCM was examined with immunofluorescence staining. Disinfected coverslips were positioned in six‐well plates, and OCCM was seeded in the plates at 3 × 10^5^ per well. After 24 hours, the cells were fixed with 4% paraformaldehyde for 10 minutes. The cells were treated with 0.1% Triton X‐100 in PBS for 15 minutes, rinsed with PBS and blocked with 5% BSA at 37°C for 1 hour. Afterwards, the cells were incubated in a rabbit anti‐YAP primary antibody dilution buffer (1:100; CST) overnight at 4°C. The cells were rinsed with PBS and then incubated with Dylight 549‐conjugated secondary antibodies (Thermo Fisher) for 1 hour. DAPI (Beyotime) was used to stain the nuclei for 10 minutes, and then, the cells were covered with an antifluorescence quenching reagent (Beyotime).

The mouse apical periodontal tissue sections were treated accordingly and incubated in a rabbit anti‐YAP primary antibody dilution buffer (1:100; CST). Subsequent steps for sections were the same as those for OCCM growing on the coverslips.

p65 protein localization was examined similarly. After growing on the coverslips for 24 hours, OCCM in the random half of the plates of each cell type was treated with 10 ng/mL TNF‐α for 30 minutes, and the cells in all the plates were fixed with 4% paraformaldehyde for 10 minutes. After being treated with Triton X‐100 and blocked, the cells were incubated in a rabbit anti‐p65 primary antibody dilution buffer (1:400; CST) overnight at 4°C. The remaining steps were the same as those of YAP localization. Staining was viewed under a fluorescence microscope (Zeiss), and micrographs were taken.

### 2.5 Lentivirus package and cell transduction

PLX304, YAP, pLKO and shYAP lentiviral particles were produced by the triple transfections of 293e cells (Invitrogen) by using psPAX2 and pMD2.G with one of the vectors pLX304, YAP‐V5 in pLX304, pLKO and shYAP in pLKO. OCCM‐30 was incubated separately with the collected lentiviral particles with 5 µg/mL polybrene in growth medium for 6 hours. Then, the medium was replaced with selective medium containing 1 µg/mL blasticidin or 2 µg/mL puromycin. After 10 days, transduction became stable because the cells seldom died in the selective medium. The YAP expression in transduced cells was analysed using Western blot.

### 2.6 Real‐time reverse transcription polymerase chain reaction (RT‐PCR)

Total RNA was extracted from single‐layer cells with TRIzol (Invitrogen) according to the manufacturer's instructions. First‐stand cDNA was reverse transcribed from 1 µg total RNA by using the PrimeScript First‐Strand cDNA Synthesis Kit (Takara) following to the manufacturer's protocol. RT‐PCR was performed in triplicate by using the SYBR Premix Ex Taq II kit (Takara) on a QuantStudio™ 6 Flex Real‐time PCR System (Applied Biosystems). Relative mRNA expression was calculated using the 2^−ΔΔ^ *^C^* ^t^ method, and housekeeping gene β‐actin was chosen as the internal control gene. The sequences of primers used are listed in Table 1.

**Table 1:** Primer sequences for qRT‐PCR

Gene	Forward primer (5′‐3′)	Reverse primer (5′‐3′)
ALP	TGTGGAATACGAACTGGATGAG	ATAGTGGGAATGCTTGTGTCTG
RUNX2	CCCAGCCACCTTTACCTACA	TATGGAGTGCTGCTGGTCTG
OCN	CTGACAAAGCCTTCATGTCCAA	GCGCCGGAGTCTGTTCACTA
β‐actin	GGCTGTATTCCCCTCCATCG	CCAGTTGGTAACAATGCCATGT

### 2.7 Western blot analysis

OCCM‐30 single‐layer cells were lysed with RIPA buffer (Beyotime) containing phosphatase inhibitors (Roche) and PMSF (Beyotime). Then, lysate was centrifuged, the supernatant was collected, and the total protein concentration was determined using the BCA Protein Assay Kit (Beyotime). Forty micrograms of total protein from each group diluted in loading buffer (Beyotime) was boiled at 95°C for 10 minutes, separated by electrophoresis in 10% SDS polyacrylamide gel (Servicebio) and wetly transferred to a PVDF membrane (Millipore). The membrane was subsequently blocked with 5% non‐fat milk for 1 hour. Afterwards, the membrane was incubated at 4°C overnight with the following primary antibodies: anti‐YAP (1:2000; CST), anti‐OCN (1:1000; Proteintech) and anti‐p‐p65 (S536; 1:1000; CST).

In the next day, the membrane was washed three times with TBST buffer, incubated with corresponding horseradish peroxidase‐conjugated secondary antibody for 1 hour at room temperature and then washed with TBST buffer three times. Blots were generated using the ECL reagent (Advansta) and detected using X‐ray films (Kodak). The protein bands were scanned using the LiDE 110 scanner (Canon) and quantified with densitometry analysis by using the ImageJ software (National Institutes of Health). Western blot analysis was repeated at least three times.

### 2.8 ALP activity analysis and ALP staining

OCCM‐30 was seeded in 24‐well plates, cultured to reach 80% confluence, pre‐treated with 10 ng/mL TNF‐α for 30 minutes and mineralization induced for 7 days. The cells were lysed in 1% Triton X‐100 on ice for 30 minutes. The total protein concentration was dertermined using a BCA protein assay kit (Beyotime). Then, 20 µL of lysate of each well was incubated in a 96‐well plate with 100 µL of fresh solution of p‐nitrophenyl phosphate substrate at 37°C for 30 minutes. The reaction was quenched by the addition of 80 µL of 0.5 mol/L NaOH solution. The absorbance at 405 nm was measured using a microplate‐reading spectrophotometer (PowerWave XS2, BioTek). A standard curve describing the relationship between OD value and p‐NP concentration was generated with the additional examination of gradient diluted p‐NP. The produced p‐NP concentration in each sample was calculated using the standard curve, and the relative ALP activity was expressed as the percentage in produced p‐NP concentration per unit time per mg protein.

For ALP staining, the cells were seeded in six‐well plates, treated and mineralization induced as those for ALP activity analysis. ALP staining was performed using a BCIP/NBT alkaline phosphatase colour development kit (Beyotime Institute of Biotechnology). Pictures were taken using a light microscope and a camera.

### 2.9 Alizarin red staining

After being pre‐treated with 10 ng/mL TNF‐α for 30 minutes and mineralization induced for 14 days, the cells underwent Alizarin red staining. The cells were fixed with 4% paraformaldehyde for 15 minutes, washed with distilled water, stained with 2% Alizarin red (pH of 4.2) for 15 minutes at room temperature and rinsed with distilled water. Photographs were taken with a camera. Besides, the stains were desorbed in 10% cetylpyridinium chloride (Sigma) for 1 hour. Two hundred microlitres of the solution was collected, placed in 96‐well plates and read at 590 nm using a spectrophotometer. The results were normalized to total protein concentration.

### 2.10 Dual‐luciferase assay

The lentivirally transduced cells, including OCCM/pLX304, OCCM/YAP, OCCM/pLKO and OCCM/shYAP, were seeded at 8 × 10^4^ cell per well into 24‐well plates. After 24 hours, Lipofectamine 2000 (Invitrogen) was used to cotransfect the cells with the reporter plasmid pNF‐κB‐luc and internal control plasmid pRL‐TK (Promega, Beijing, China) at a ratio of 10:1. After 6 hours, the medium was changed into DMEM with 10% FBS and 10 ng/mL TNF‐α, and the cells were incubated for 30 minutes before lysis. Luciferase activity was measured according to the manufacturer's instructions.

### 2.11 Statistical analysis

Statistical analysis was performed using the GraphPad Prism software. The differences between groups were examined with Student's *t* test. The level of significance was set at *P* = .05.

## 3 RESULTS

### 3.1 YAP protein expression and localization

**Figure 1:**
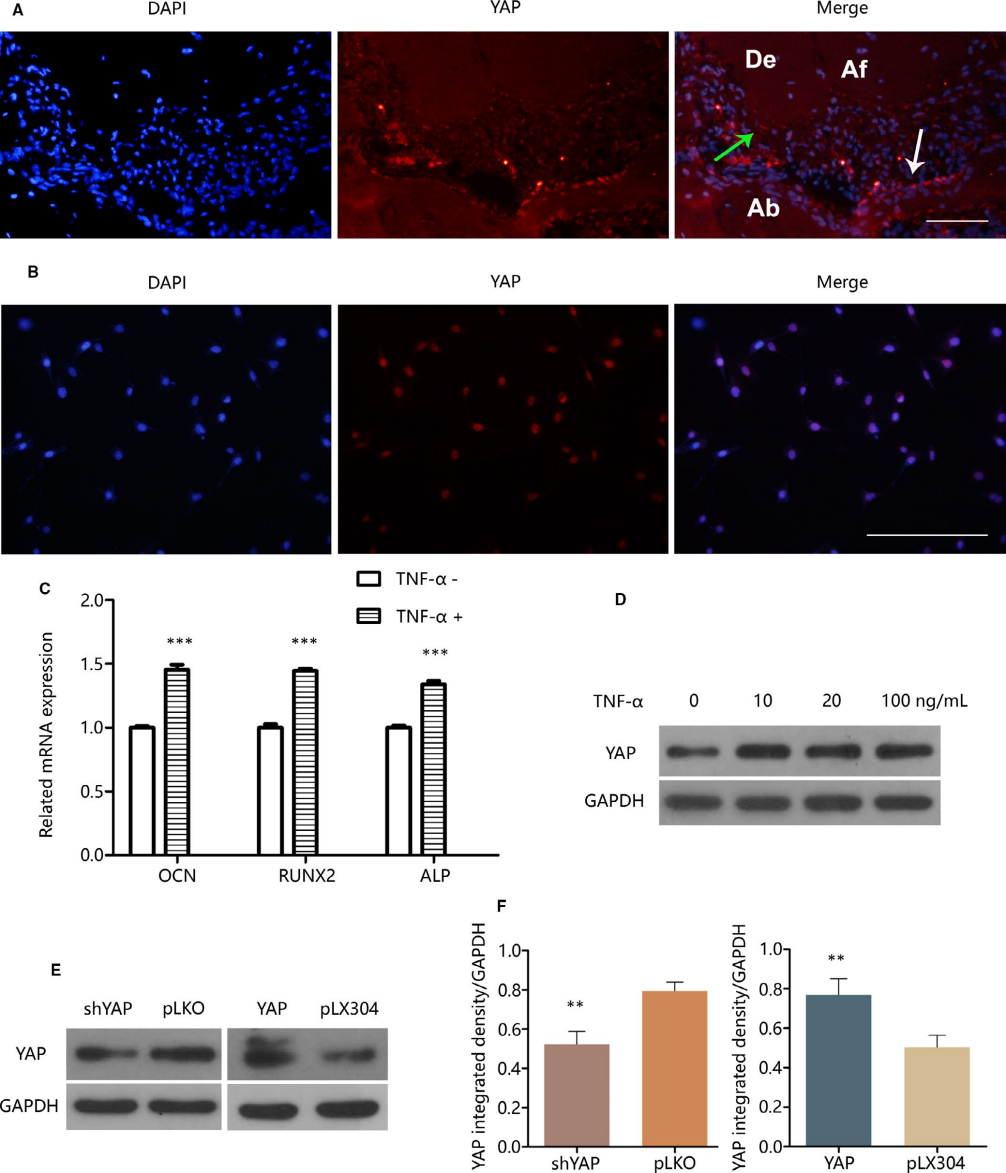
Inflammatory cytokine TNF‐α treatment can promote OCCM mineralization and increase YAP expression. A, Fluorescent staining showed that YAP was expressed in the periapical tissue of the simulated mouse apical periodontitis. De = dentin, Af = apical foramen, Ab = alveolar bone. Arrows show the locations of positive YAP. B, Fluorescent staining showed that YAP was mainly located in OCCM nuclei. C, Cementogenesis‐related gene expression was examined with qPCR after transient 10 ng/mL TNF‐α treatment for 30 min and then mineralization induced for 7 d. D, YAP protein expression was determined with Western blot after treatment with or without TNF‐α for 24 h. E and F, Western blot and quantification with ImageJ showed the YAP expression of lentivirally transduced OCCM‐30. Scale bar = 200 µm. **P < .01, ***P < .001

The immunofluorescent micrographs taken from mouse tissue sections showed that YAP was expressed in the apical cementum of the induced apical periodontitis (Figure 1A). The micrographs taken from cultured cells showed that YAP was expressed in OCCM and located in the nuclei (Figure 1B).

### 3.2 Promoted OCCM mineralization and elevated YAP expression by inflammatory cytokine TNF‐α treatment

It was then investigated whether the mineralizing potential of OCCM would be affected by TNF‐α treatment of limited duration. After treatment with 10 ng/mL TNF‐α for 30 minutes and then mineralization induction for 7 days, mineralization related genes ALP, RUNX2 and OCN were all surprisingly up‐regulated compared with those in OCCM that were not treated with TNF‐α (Figure 1C).

Then, we evaluated YAP expression after TNF‐α treatment. After OCCM reached 80% confluence, TNF‐α was added into the growth medium at various concentrations (10, 20, and 100 ng/mL) and cell culture was continued for the next 24 hours. YAP protein expression was increased in TNF‐α‐treated OCCM‐30 compared with the control group (0 ng/mL TNF‐α) (Figure 1D). To investigate YAP function, we used lentiviral vectors to overexpress or knockdown YAP in OCCM. Western blot examination showed the successful establishment of YAP overexpression and knockdown OCCM cell lines (Figure 1E and F).

### 3.3 Mineralization promotion by YAP overexpression after TNF‐α treatment

**Figure 2:**
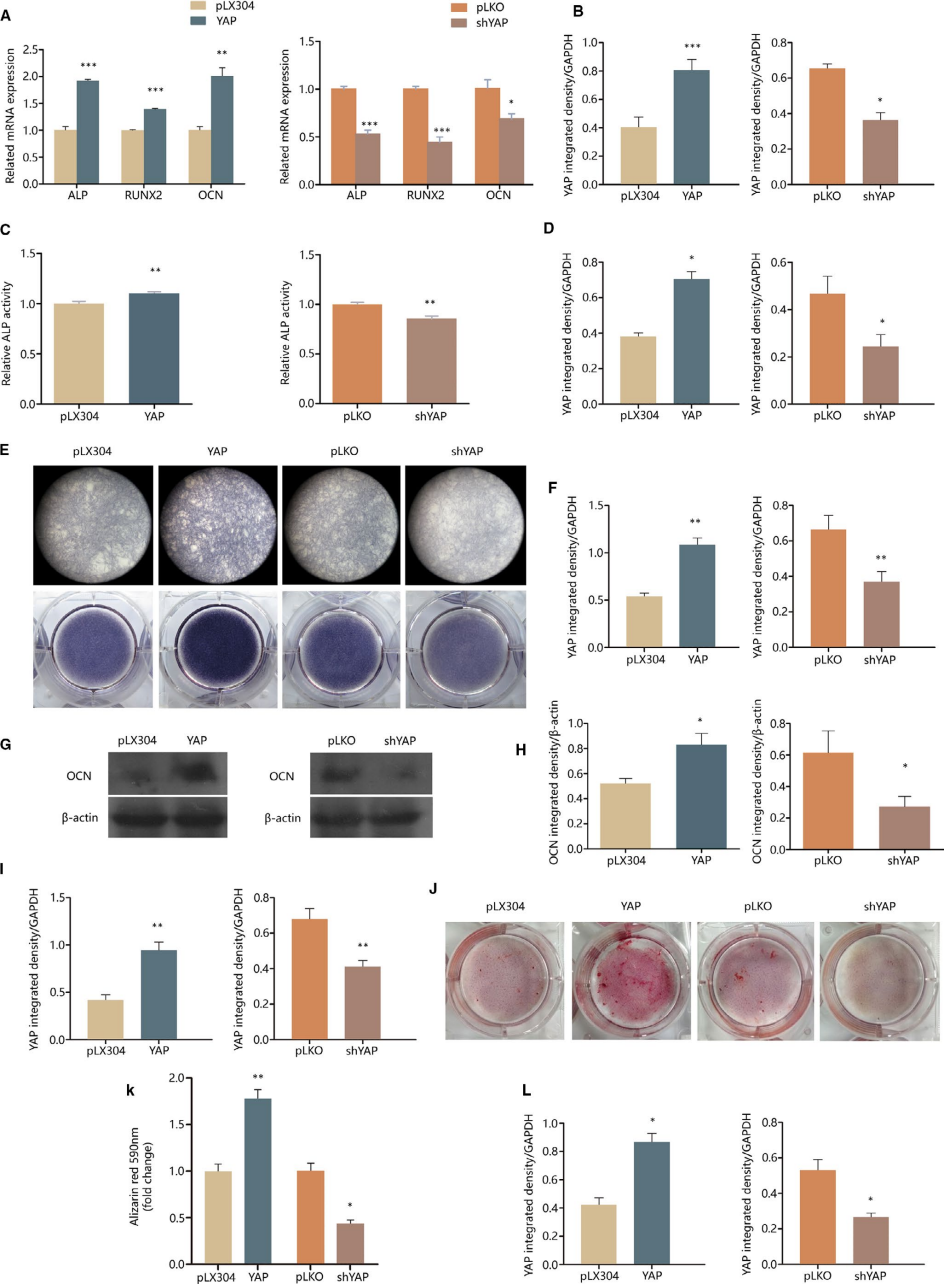
Effect of YAP on OCCM‐30 mineralization after 30 min of 10 ng/mL TNF‐α treatment. For each test, YAP protein expression in the tested transgenic cell lines was confirmed with Western blot and quantified with ImageJ (n = 3). A and B, The transcriptional level of the mineralization‐related genes ALP, RUNX2 and OCN on day 7 were determined by qPCR. The 2^−ΔΔ^ *^C^* ^t^ values were used for statistical analysis (n = 3). C and D, ALP activity was measured on day 7 of mineralization induction (n = 6). E and F, ALP staining was performed on day 7 of mineralization induction. G, H and I, OCN protein expression on day 7 of mineralization induction was examined with Western blot and quantified with ImageJ (n* = *3). J, K and L, Alizarin red staining was performed on day 14 and quantified (n* = *3). Histogram results are shown as mean ± SEM.*P < .05, **P < .01, ***P < .001

The intriguing promoted mineralization by TNF‐α treatment leads us to figure out whether YAP still plays a positive role in OCCM mineralization after short‐term TNF‐α treatment as it does in OCCM without TNF‐α treatment, which was demonstrated previously.^6^ After pre‐treatment with 10 ng/mL TNF‐α for 30 minutes, YAP overexpressing OCCM showed promoted mineralization compared with control cells, and YAP knockdown OCCM exhibited suppressed mineralizing activity (Figure 2). Alizarin red staining showed that YAP overexpressing OCCM had enhanced mineralizing phenotype compared with the control, and YAP knockdown OCCM had compromised mineralizing phenotype (Figure 2J‐L). Western blot results showed that the protein expression of mineralization related gene OCN was up‐regulated in YAP overexpressing OCCM and down‐regulated in YAP knockdown OCCM (Figure 2G‐I). The transcriptional levels of ALP, RUNX2 and OCN were similarly regulated (Figure 2A and B). ALP activity and ALP staining were affected similar to ALP mRNA (Figure 2C‐F).

### 3.4 NF‐κB pathway activity inhibition by YAP

**Figure 3:**
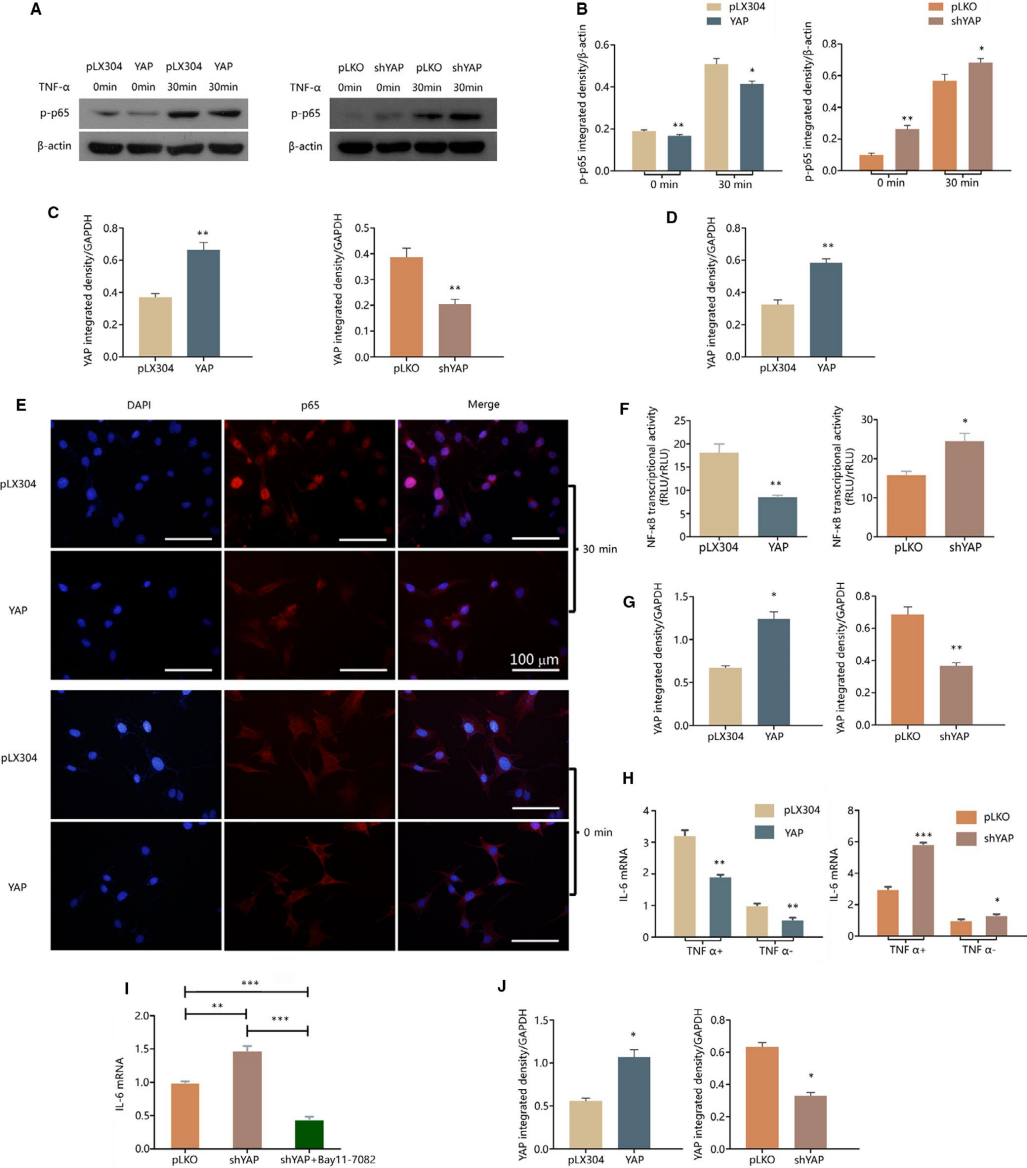
Effect of YAP on the NF‐κB pathway activity of OCCM‐30. For each test, YAP protein expression in the tested transgenic cell lines was confirmed with Western blot and quantified with ImageJ. A‐C, The protein of phosphorylated p‐65 was examined with Western blot in lentivirally transduced OCCM‐30 with or without 10 ng/mL TNF‐α treatment for 30 min; the results were quantified with ImageJ. D and E, Immunofluorescence staining showed that the NF‐κB‐p65 nucleus translocation was inhibited in YAP overexpressing OCCM‐30, compared with control OCCM, whether or not treated with 10 ng/mL TNF‐α for 30 min. Scale bar = 100 µm. F and G, After the cells were treated with 10 ng/mL TNF‐α for 30 min, NF‐κB transcriptional activities in transduced OCCM‐30 were detected with dual‐luciferase reporter system. H and J, After the cells were treated with or without 10 ng/mL TNF‐α for 30 min, IL6 mRNA expression was quantified by qRT‐PCR in transduced OCCM‐30. I and J, The IL6 mRNA level in OCCM/pLKO, OCCM/shYAP and Bay 11‐7082 pre‐treated OCCM/shYAP, after 30 min of treatment with 10 ng/mL TNF‐α. *P < .05, **P < .01, ***P < .001; n = 3

YAP expression was increased by TNF‐α treatment (in Figure 1D), thereby indicating that YAP may play a role in inflammation‐related pathway in OCCM. Hence, the regulation of YAP on NF‐κB pathway activity, which is a major inflammatory pathway, was investigated. The results showed that the NF‐κB pathway activity in OCCM was inhibited by YAP (Figure 3). Western blot analysis showed that regardless of TNF‐α addition, YAP overexpression inhibited, and YAP knockdown promoted the phosphorylation level of p65, which is an indicator of the NF‐κB pathway activity (Figure 3A‐C). Immunofluorescence staining showed that YAP overexpression inhibited the nuclear translocation of p65 in OCCM (Figure 3D and E). Luciferase assay showed that after treatment with 10 ng/mL TNF‐α for 30 minutes, YAP overexpression suppressed, and YAP knockdown elevated NF‐κB transcriptional activity (Figure 3F and G). The transcriptional level of IL6, as an indicator of NF‐κB pathway activity, was down‐regulated in YAP overexpressing OCCM and up‐regulated in YAP knockdown OCCM (Figure 3H and J). The promotion of IL6 mRNA by YAP knockdown in OCCM treated with TNF‐α was rescued by pre‐treatment with NF‐κB pathway‐specific inhibitor, Bay11‐7082 (Figure 3I and J).

### 3.5 NF‐κB pathway inhibition partially rescuing YAP knockdown decreased OCCM mineralization ability

**Figure 4:**
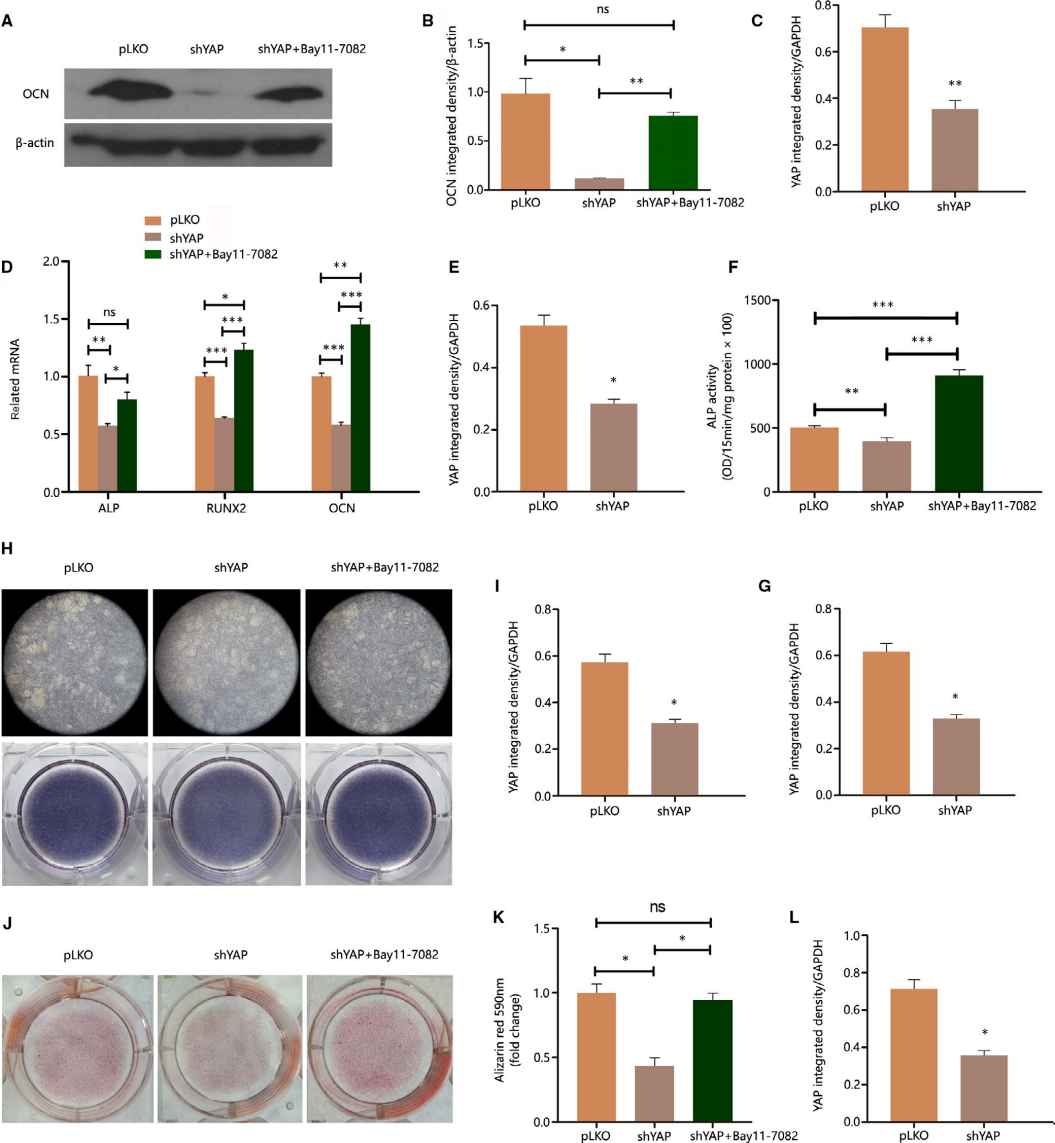
YAP promotes TNF‐α–treated OCCM‐30 mineralization partly by inhibiting NF‐κB pathway. For each test, YAP protein expression in the tested transgenic cell lines was confirmed with Western blot and quantified with ImageJ (n = 3). A‐C, OCN and β‐actin immunoblot of OCCM/pLKO, OCCM/shYAP and Bay 11‐7082 pre‐treated OCCM/shYAP, after 10 ng/mL TNF‐α treatment for 30 min and mineralization induction for 7 d; the results were quantified with ImageJ. D and E, ALP, RUNX2 and OCN transcriptional levels were determined with qPCR in OCCM/pLKO, OCCM/shYAP and Bay 11‐7082 pre‐treated OCCM/shYAP, after 10 ng/mL TNF‐α treatment for 30 min and mineralization induction for 7 d (n = 3). F and G, ALP activity was measured after 10 ng/mL TNF‐α treatment for 30 min and mineralization induction for 7 d (n = 6). H and I, ALP staining was conducted after 10 ng/mL TNF‐α treatment for 30 min and mineralization induction for 7 d. J‐L, Alizarin red staining was performed and quantified on day 14 of mineralization (n* = *3). Histogram results are shown as mean ± SEM.*P < .05, **P < .01, ***P < .001

The results above showed that the altered mineralization of transgenic OCCM may correlate with YAP affected NF‐κB pathway activity. To elucidate the role of NF‐κB pathway, we performed rescue experiments with NF‐κB pathway‐specific inhibitor Bay11‐7082. Serving as the rescue group, shYAP OCCM dishes were pre‐treated with 3 µmol/L Bay11‐7082 for 30 minutes after reaching 80% confluence. Then, the rescue group along with pLKO and shYAP OCCM of the same cell density were treated with 10 ng/mL TNF‐α for 30 minutes, and subsequently mineralization induced and analysed. The rescue experiment results showed that the effect of YAP on the mineralization of TNF‐α‐treated OCCM was exerted partly through regulating the NF‐κB pathway activity (Figure 4). After pre‐treatment with Bay11‐7082, the expression of mineralization‐related genes, ALP activity and calcium deposits were all rescued in YAP knockdown OCCM, which exhibited inhibited mineralization previously.

## 4 DISCUSSION

In this study, YAP was found to inhibit the NF‐κB pathway activity and transcriptional level of IL6 in OCCM after TNF‐α treatment. And after TNF‐α treatment, YAP was revealed to promote cementogenesis via suppressing the NF‐κB pathway. This finding provides molecular insight into cementum regeneration under inflammatory cytokine challenge of limited duration, which may aid in understanding the mechanism of periodontal regeneration.

YAP is a transcription coactivator that is involved in cell proliferation, organ size determination and tissue repair. Figure 1D shows that YAP expression was up‐regulated in OCCM after TNF‐α treatment, thereby suggesting that YAP may play a role in inflammatory response.

The osteogenesis process is similar to that of cementogenesis, and YAP has been reported to promote osteogenesis of other cells types. Zhang et al demonstrated that the osteogenesis of MC3T3‐E1 cells enhanced by topography requires the participation of YAP.^13^ Kegelman et al reported that YAP and its paralog TAZ promote mice bone development.^14^ As for cementogenesis, YAP has been shown to have a positive effect through regulating the BMP/Smad and Erk1/2 pathways.^7^ In inflammatory microenvironment, it is important how YAP influences NF‐κB activity which is often found detrimental to cell mineralization. Gao found that shYAP overexpression increased the transcriptional activity of NF‐κB in 293T cells.^15^ However, YAP interacts with p65 in breast cancer cells and promotes the expression of p65 target genes.^16^ In cementoblast, the effect of YAP on the NF‐κB activity remains unclear. This effect is supposed to be associated with how YAP regulates cementogenesis under inflammation challenge.

In the present study, the results showed that YAP suppressed the NF‐κB pathway activity and the transcription of pro‐inflammatory cytokine IL6. NF‐κB pathway more often plays a negative role in cell mineralization. Swarnkar reported that constitutive NF‐κB activation in mice impairs skeletal development and perturbs maturation of calvarial osteoblasts.^10^ Chang demonstrated that activated NF‐κB induces Smurf1 and Smurf2, thereby promoting the degradation of β‐catenin in mesenchymal stem cells (MSCs) and inhibiting their osteogenesis.^9^ When NF‐κB is inhibited in MSCs, bone formation is enhanced by MSC‐mediated osteogenesis. Wang reported that the dental pulp stem cells (DPSCs) obtained from oestrogen‐deficient mice have a more activated NF‐κB activity and impaired osteogenic potential, and the impaired osteogenic potential is rescued by the treatment of NF‐κB specific inhibitor.^11^ In the present study, Figure 4 shows that the affected cementogenetic potential and the expression of related genes in shYAP OCCM were rescued by pre‐treatment of NF‐κB specific inhibitor Bay11‐7082. This result suggested that NF‐κB plays a negative role in OCCM cementogenesis. The results in Figure 3 confirmed the inhibition of NF‐κB by YAP, which was consistent with the promotion of cementogenesis by YAP, as demonstrated in Figure 2. In this study, 30 minutes of TNF‐α treatment showed a promotion instead of cementogenesis inhibition. This condition may be due to other pathways that we did not investigate here except NF‐κB. TNF‐α inhibits cementoblast mineralization when the treatment lasts.^17^ Hence, inflammation influences cementogenesis also in an intricate manner, thereby exhibiting varied effects in different contexts. This finding prompts further investigation into the mechanism underlying how limited inflammation promotes cementogenesis.

Considering that YAP inhibits NF‐κB activity and promotes cementogenesis, whether NF‐κB inhibition rescues the impaired cementogenesis of shYAP cells was investigated. The results showed that the affected cementogenesis was rescued by pre‐treatment of NF‐κB specific inhibitor, which indicated that the effect of YAP on cementogenesis was exerted partly by regulating the NF‐κB pathway.

## CONFLICT OF INTEREST

The authors confirm that there are no conflicts of interest.

## AUTHOR CONTRIBUTIONS

Lu Zhang and Hualing Sun designed and performed the experiments, analysed the data and also wrote the paper; Jing Zhang provided study material; Fangfang Song designed the experiments; Liyuan Huang contributed to data analysis; Zhengguo Cao provided essential study material; Cui Huang designed the experiments, revised the paper and provided financial support. All authors read and approved the manuscript.

## Data Availability

The data that support the findings of this study are available from the corresponding author upon reasonable request.
